# Long-term follow-up results of medial opening wedge high tibia osteotomy with a pre-countered non-locking steel plate

**DOI:** 10.1007/s00402-021-03927-8

**Published:** 2021-05-11

**Authors:** Simo S. A. Miettinen, Hannu J. A. Miettinen, Jussi Jalkanen, Antti Joukainen, Heikki Kröger

**Affiliations:** 1 Department of Orthopaedics, Traumatology, and Hand Surgery, Kuopio University Hospital, P.O. Box 1777, 70211 Kuopio, Finland; 2 Faculty of Health Sciences, University of Eastern Finland, Yliopistonranta 1, 70210 Kuopio, Finland

**Keywords:** Opening wedge high tibial osteotomy, Survival analysis, Adverse events, Survival, Knee surgery, Osteoarthritis

## Abstract

**Introduction:**

This retrospective study investigated the long-term follow-up results of medial opening wedge high tibial osteotomy (MOWHTO) with a pre-countered non-locking steel plate implant (Puddu plate = PP) used for medial knee osteoarthrosis (OA) treatment.

**Materials and methods:**

Consecutive 70 MOWHTOs (66 patients) were performed between 01.01.2004 and 31.12.2008 with the mean follow-up time of 11.4 (SD 4.5; range 1.2–16.1) years. The Kaplan–Meier survival analysis was used to evaluate the cumulative survival of the implant in terms of age (< 50 years old and ≥ 50 years old) and gender. Adverse events were studied and Cox regression analysis was used to evaluate risk factors [age, gender, body mass index (BMI), preoperative mechanical axis, severity of OA, use of bone grafting or substitution and undercorrection of mechanical axis from varus to valgus] for revisions.

**Results:**

The estimates for the cumulative survival with no need for TKA after MOWHTO were 86% at 5 years, 67% at 10 years and 58% at 16.1 years (SE 0.6, CI 95% 11.1–13.5). A total of 33/70 (47%) adverse events occurred and 38/70 (54%) knees required some revision surgery during the follow-up. Cox regression did not show any statistically significant risk factors for revision.

**Conclusions:**

The PP has feasible MOWHTO results with a cumulative survival of 67% at 10 years with no need for conversion to TKA. Many adverse events occurred and revision rate due to any reason was high. Age or gender did not have statistically significant differences in terms of survival.

**Supplementary Information:**

The online version contains supplementary material available at 10.1007/s00402-021-03927-8.

## Introduction

High tibial osteotomy (HTO) has been advocated for the treatment of isolated medial compartment osteoarthritis (OA) of the knee since the 1970s [1–4]. The HTO techniques have evolved and in the late 1990s and early 2000s, medial opening wedge HTO (MOWHTO) with internal fixation was popularized [5–7]. In the late 1990s, Giancarlo Puddu published a simple, reproducible technique based on the author’s own implant design [8, 9]. This implant is known as the Puddu plate (PP) (Arthrex^®^) which is a pre-countered non-locking steel plate that has a metallic block to provide stabilization and determine the size of the opening wedge osteotomy [8, 9].

Although HTO is known to be a successful operation, OA often progresses over the years, necessitating total knee arthroplasty (TKA). To this, a recent meta-analysis showed that patients who undergo a conversion of HTO to TKA have similar 10-year survival rates as patients who undergo primary TKA but these conversion patients had a higher infection rate and somewhat poorer range of motion [10]. A lot of variables, such as age, gender, smoking, BMI, type of plate (non-locking or locking), preoperative radiological findings, surgeon experience, surgical technique (closed-wedge, open-wedge or external fixator), use of bone grafting (autologous or allograft) and use of bone substitutes are affecting the survival of MOWHTO [5, 7, 11–15].

The purpose of this study was to evaluate the long-term survival of the patients who underwent MOWHTO with the PP as a treatment for the medial compartment OA of the knee and to evaluate reasons and risk factors for revisions and later conversion to TKA. Special study interests were to compare survival between the genders and the patients aged < 50 years old and ≥ 50 years old.

## Materials and methods

### Study design and patients

A retrospective analysis was performed for all patients who underwent MOWHTO with PP for OA at Kuopio University Hospital (KUH) during the study period (from 01.01.2004 to 31.12.2008). A total of 72 knees (68 patients) were consecutively operated on during the study period, and of these, 70 knees (66 patients) were included. Two patients were excluded from the study due to technical errors of the osteotomy, which led to revision surgeries < 3 months from the index HTO surgery. All patients initially presented with symptoms of unicompartmental medial overload originating from varus malalignment of the leg with incipient medial femorotibial OA. The follow-up ended on 30.06.2020 or revision to TKA surgery. Patients’ current knee status at the end of follow-up was checked to find out that they did not have had any revision operations in another hospital.

**Fig. 1:**
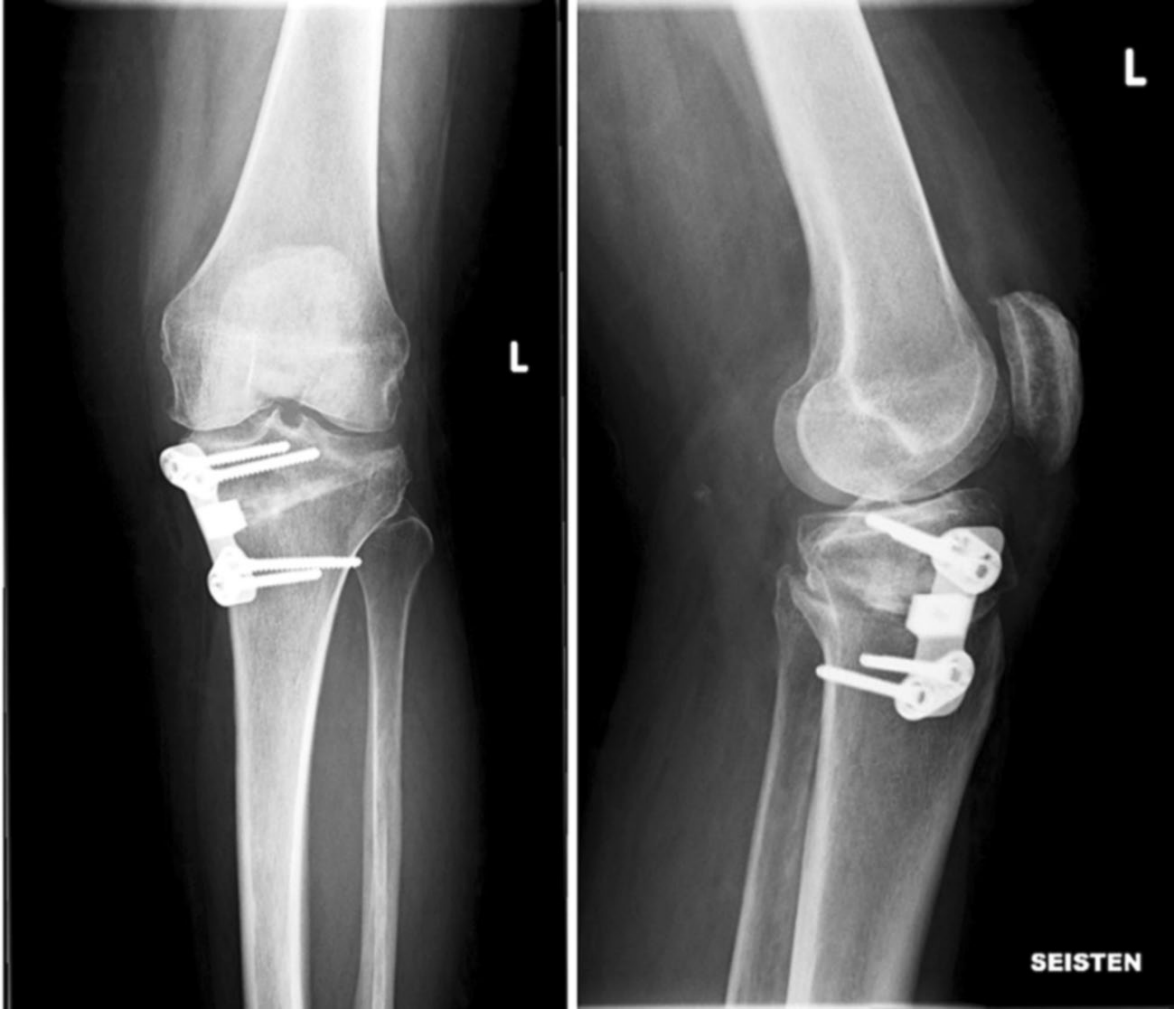
Post-operative (12 weeks) anteroposterior and medio-lateral knee radiographs of the Puddu plate

A pre-countered non-locking steel plate with incorporated spacer known as a PP was used (Arthrex^®^, Naples, Florida, USA) (Fig. 1). Patients’ data were collected from the KUH medical register, including demographic data [gender, age, operation side, and body mass index (BMI)] and surgical details (operation time, use of bone graft or bone substitution). Intraoperative complications, adverse events and conversion to total knee arthroplasty were evaluated. Adverse events were defined as minor (no need for revision surgery) and major (revision surgery due to any reason but not conversion to TKA). Implant removal was considered as a major adverse event. Revised patients’ demographic data and radiological measures were compared to non-revised patients’ data. Time from the revision surgery due to pain or implant irritation to later TKA was evaluated.

### Radiological analysis

Radiographic grading of the knee OA was done according to the Kellgren–Lawrence (K–L) scale and cartilage defects were evaluated [16, 17]. In this study, criteria for MOWHTO were: 1. medial OA compartment K–L 1–3, and 2. K–L 0–1 OA for patellofemoral (PF) or lateral tibiofemoral joints in radiographs or 3. mild to moderate cartilage defects in medial OA compartment in perioperative or previous arthroscopy. Patients with significant OA changes in lateral compartment and/or PF joint in radiographs or in perioperative arthroscopy were not suitable for MOWHTO.

**Fig. 2:**
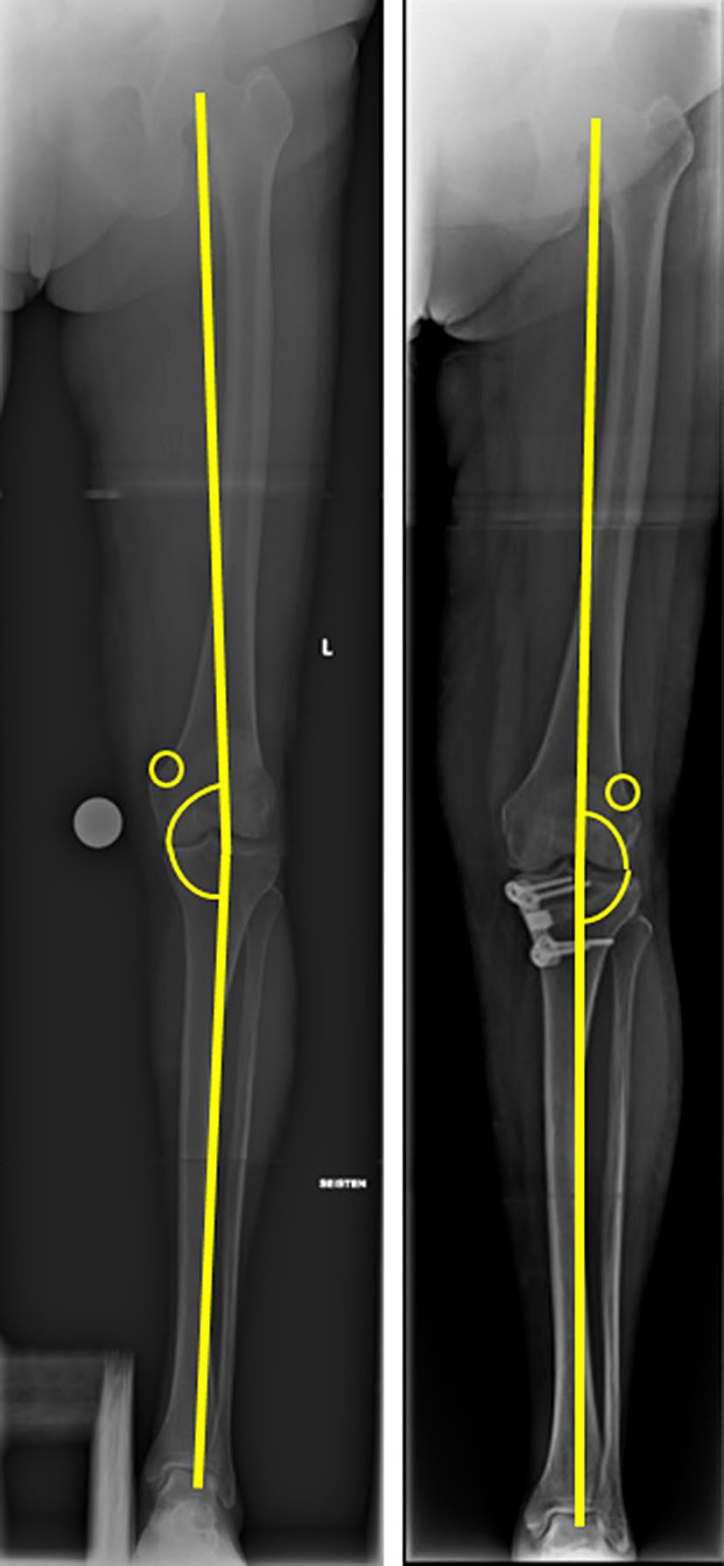
Pre- and postoperative radiographs of the mechanical axes of the lower extremity. Hip–knee–ankle angle is coloured yellow and it is varus in preoperative radiograph and valgus in postoperative, respectively

Radiological assessments were made from anteroposterior and lateral full weight-bearing radiographs. The preoperative and postoperative (6 and/or 12 weeks) mechanical axes of the lower extremity (hip–knee–ankle angle = HKA) (Fig. 2) and medial proximal tibia angle (MPTA) were measured via long-leg weight-bearing anteroposterior native radiographs. A normal HKA is 1.0°–1.5° of varus angulation (180°, ± 3°) and normal MPTA is 87° (range 85°–90°) [3, 18]. Change of HKA from varus to valgus was evaluated, as well as change of MPTA. The posterior proximal tibial angle (PPTA) and its change was measured pre- and postoperatively. A normal PPTA is 81° (range 77°–84°) [3]. The size of the opening wedge was measured from the postoperative radiographs. Only patients with varus HKA, normal MPTA and normal lateral distal femoral angle (LDFA) were operated on with MOWHTO. Preoperative planning was made by adjusting the HKA of the lower extremity to pass through 62–62.5% of the tibial plateau from the proximal tibial edge, the so-called ‘Fujisawa point’ [19]. The aim of the HKA correction was to change varus to valgus angle.

### Operative techniques

Six experienced consultant orthopaedic surgeons performed operations. All patients had epidural or spinal anaesthesia, and some patients had additional general anaesthesia. Preoperative intravenous antibiotic prophylaxis (cefuroxime 1.5 g or clindamycin 600 mg) was regularly used. Open wedge osteotomy was performed on all patients medially approximately 3–4 cm below the joint line. The pes anserinus tendons were dorsally retracted, and the medial collateral ligament was released distally if needed. Under fluoroscopic guidance, a guide wire was advanced medially from 1 to 2 cm distal to the level of the joint up to the lateral cortex [4]. A monoplanar osteotomy was performed by using an oscillating saw, and care was taken to avoid violation of the lateral cortex. The osteotomy was gradually opened by blades to preserve a bony bridge laterally in line with the joint line. In this study, monoplanar osteotomy was commonly used during the study period even though there was already by that time some postulations that biplanar osteotomy would increase the primary stability of the bone-implant construct in the sagittal plane [19]. The osteotomy wedge gap was filled by an iliac crest bone graft (autograft) or freeze-dried corticocancellous structural grafts (allograft) and, in some cases, with bone substitutions (Actifuse^™^, Baxter Healthcare, Deerfield IL, USA and ChronOS^™^, Synthes, Umkirch, Germany). The indication for use of bone graft or a substitute was not possible to study retrospective as in medical records it usually was not mentioned. Limited weight-bearing was allowed for first 6–8 weeks postoperatively, and after that, gradual full weight-bearing was allowed. Full range of motion was allowed for all patients after MOWHTO. If lateral hinge fracture occurred, a non-weight bearing was instructioned for 6 weeks.

### Outcome measures

The primary outcome measure was survival without a need for revision to TKA. The secondary outcome was major adverse event including plate removal.

### Statistical methods

Kaplan–Meier analysis and the log-rank test were used to study postoperative survival where the endpoint was revision surgery for any reason and conversion to TKA. Comparison of continuous data was carried out using a Mann–Whitney *U* test. For categorical data, a chi-square test was used. The independent samples *t *test was used for parametric data comparison. Cox regression models were used in both univariate (single risk factors) and multivariate (combinations of risk factors) analyses to evaluate risk factors for revision and conversion to TKA. The variables analysed were age, gender, BMI, preoperative mechanical limb axis and bone graft or bone substitution, preoperative grade of OA and undercorrection of HKA. Two-tailed *p* values are reported. All *p* values  ≤ 0.05 were considered statistically significant. All data were analysed using SPSS (SPSS Inc., Chicago, IL, USA. Ver 27.0.0, IBM).

## Results

The mean follow-up time was 11.4 (SD 4.5; range 1.2–16.1) years. No patients were lost to follow-up. The mean age of the patients was 50.4 (SD 8.0; range 23.3–63.3) years. The mean BMI was 30.8 (SD 5.4; range 22.4–41.7) kg/m^2^, which means obesity (BMI > 30.0 kg/m^2^). The mean operation time was 78 (SD 19.0; range 34–127) minutes. Bone grafting and/or bone substitution was used in all knees (Tables 1, 2). Radiological analyses were done to all knees pre- and postoperatively and between group (revised vs. non-revised) comparisons were done in terms of HKA, MPTA, PPTA and osteotomy gap but no statistically significant differences were found (Tables 1, 2). Varus HKA changed to valgus in 47/59 (78%) of the knees while in 12/59 knees it stayed in varus or was straight which means that HKA was undercorrected. Of these undercorrected patients, 6/12 had a revision with a mean time of 3.6 years from the index operation while in those whose post-op HKA was in valgus, the mean time to revision was 4.8 years (*p* = 0.49). Preoperative HKA radiographs were not available for this study in 11 knees.

**Table 1:** Demographic data of the operated patients, operational data and radiological measurements

	*n* = 70 *n* (%)
Gender	
Male	46 (70)
Female	24 (30)
Operation side	
Right	35 (50)
Left	35 (50)
Kellgren–Lawrence classification	
1	18 (26)
2	39 (56)
3	13 (19)
Type of bone and substitution grafting	
Autograft from iliac crest	30 (43)
Actifuse bone substitute	22 (31)
Allograft bone	11 (16)
Allograft bone and autograft bone	6 (9)
Autograft from iliac crest and Chronos bone substitute	1 (1)

	Mean (SD, range)
HKA (°)	
Preoperatively (varus)	5.8 (2.9, 0.8–16.7)
Postoperatively (valgus)	− 1.8 (3.9, − 10.0–13.0)
Change of axis angle*	7.7 (4.2, − 3.0–18.1)
MPTA (°)	
Preoperatively	86.8 (2.5, 78.5–92.1)
Postoperatively	91.4 (4.1, 79.2–99.8)
Change of MPTA angle*	4.6 (4.0, − 9.7–12.7)
PPTA (°)	
Preoperatively	82.4 (4.5, 0.0–21.6)
Postoperatively	78.9 (4.8, 2.1–30.6)
Change of slope angle*	3.7 (4.4, − 5.7–15.3)
Osteotomy gap opening (mm)	11.0 (2.2, 5.0–15.3)

*HKA* hip–knee–ankle angle; *MPTA *medial proximal tibia angle; *PPTA *posterior proximal tibial angle

*Comparison of pre- and postoperative angles were done and there were no statistically significant differences (*p* = n.s.)

**Table 2:** Comparison of revised patients to non-revised patients with any osteotomy gap filling and radiological comparison of non-revised patients to revised patients

	No revision (*n* = 32)	Revision (*n* = 38)	*p* value
	*n* (%)	*n* (%)	
Bone/substition grafting			0.09
Autograft from iliac crest	11 (34)	19 (50)	
Actifuse bone substitute	10 (31)	12 (32)	
Allograft	9 (28)	2 (5)	
Allograft bone and autograft bone	2 (6)	4 (11)	
Autograft from iliac crest and Chronos bone substitute	0	1 (3)	

	Mean (SD, range)	Mean (SD, range)	
HKA (°)			
Preoperatively	6.7 (3.3, 0.8–16.7)	5.2 (2.3, 1.6–10.8)	0.43
Postoperatively	− 1.9 (4.4, − 10.0–9.7)	− 1.3 (4.2, − 7.7–13.0)	0.40
Change of axis angle	9.0 (4.8, 0.9–18.1)	6.5 (3.6, − 3.0–15.7)	0.28
MPTA (°)			
Preoperatively	87.6 (2.2, 82.9–91.1)	85.9 (2.4, 78.5–90.0)	0.78
Postoperatively	92.7 (4.0, 79.2–99.8)	90.0 (3.9, 80.4–97.9)	0.46
Change of MPTA angle	4.8 (4.7, − 9.7–11.6)	4.4 (3.3, − 3.7–12.7)	0.30
PPTA (°)			
Preoperatively	82.6 (4.9, 0.0–21.6)	82.4 (4.3, 0.0–16.3)	0.43
Postoperatively	79.2 (5.7, 2.1–30.6)	78.7 (4.4, 3.2–22.1)	0.53
Change of slope angle	3.4 (4.8, − 5.7–16.0)	3.7 (3.9, − 4.2–11.0)	0.45
Osteotomy gap opening (mm)	11.4 (2.1, 7.5–15.0)	11.0 (2.0, 7.5–15.3)	0.31

*HKA* hip–knee–ankle angle; *MPTA* medial proximal tibia angle; *PPTA* posterior proximal tibial angle

There were 7/70 (10%) intraoperative complications, and all of them were lateral cortex fractures, which were treated conservatively with 6 weeks non-weight-bearing limitation without further related complications. A total of 33/70 (47%) adverse events occurred and 38/70 (54%) knees required some revision surgery during the follow-up (Table 3). The mean time to revision surgery for any reason was 4.6 (SD 3.9; range 0.4–14.3) years. A total of 30/70 (43%) of the knees had a conversion to TKA during follow-up, and of these, 6/70 (9%) knees had implant removal surgery first and subsequent TKA. The mean time to TKA after MOWHTO was 6.8 (SD 3.8; range 1.2–14.3) years, and the mean time from the implant removal surgery to TKA was 4.5 (SD 4.0; range 1.3–12.2) years.

**Table 3:** Adverse events, revisions and types of revision surgeries

	*n* = 70 *n* (%)
Adverse event	
No	37 (53)
Yes	33 (47)
Minor	10 (14)
Superficial infection	7 (10)
Superficial or deep venous thrombosis	2 (3)
Mild persistant pain	1 (1)
Major	23 (33)
Moderate/hard persistant pain or implant irritation	20 (29)
Anterior cruciate ligament insufficiency	2 (3)
Unhealed osteotomy	1 (1)
Revision	
No	32 (46)
Yes	38 (54)
Reason for revision	
Progression of osteoarthrosis	16 (23)
Medial knee pain/implant irritation	19 (27)
Non-union	2 (3)
Infection	1 (1)
Type of the first revision surgery	
Total knee arthroplasty	24 (34)
Implant removal	11 (16)
Implant removal and anterior cruciate ligament reconstruction	2 (3)
Implant exchange and autologous bone transfer	1 (1)

Comparison of revised and non-revised MOWHTOs was done in terms of the use of bone graft or substitution and radiological measurements (Table 2). The mean age of the revised patients at the index MOWHTO surgery was 52.7 (SD 5.7; range 40.6–63.3) years, and in the non-revision group, it was 48.8 (SD 8.9, range 23.3–59.4) years (*p* = 0.04). The Cox regression models showed that age, gender, BMI preoperative HKA, preoperative classification of OA and undercorrection of HKA were not risk factors for MOWHTO revision or conversion to TKA (Table 4). The use of bone grafting or substitution founded to be a risk factor for revision and conversion to TKA (Table 4).

**Table 4:** Cox regression univariate model was used in analyses to evaluate risk factors for revision and conversion to total knee arthroplasty (TKA)

	Revision for any reason				Conversion to total knee arthroplasty			
	HR	95% CI		*p* value	HR	95% CI		*p *value
Age	1.03	0.97	1.07	0.26	1.07	1.00	1.14	0.04
Gender								
Male	1				1			
Female	1.04	0.55	1.94	0.91	1.13	0.52	2.45	0.76
Body mass index	0.96	0.89	1.04	0.35	1.02	0.94	1.12	0.61
Preoperative mechanical axis	0.88	0.78	1.00	0.50	0.86	0.74	1.01	0.07
Type of bone and substitution grafting								
None	1				1			
Autograft from iliac crest	0.02	0.00	0.31	0.01	0.02	0.00	0.31	0.01
Actifuse bone substitute	0.01	0.00	0.23	0.01	0.02	0.00	0.31	0.01
Others	0.01	0.00	0.14	< 0.001	0.00	0.00	0.05	< 0.001
Radiographic grade of osteoarthrosis [20]								
Grade 1	1				1			
Grade 2	0.71	0.37	1.39	0.32	1.36	0.54	3.46	0.51
Grade 3	0.54	0.21	1.37	0.19	1.29	0.39	4.22	0.68
Undercorrection of mechanical axis from varus to valgus	1.22	0.50	2.93	0.66	1.21	0.42	3.52	0.73

Multivariate data not shown

**Fig. 3:**
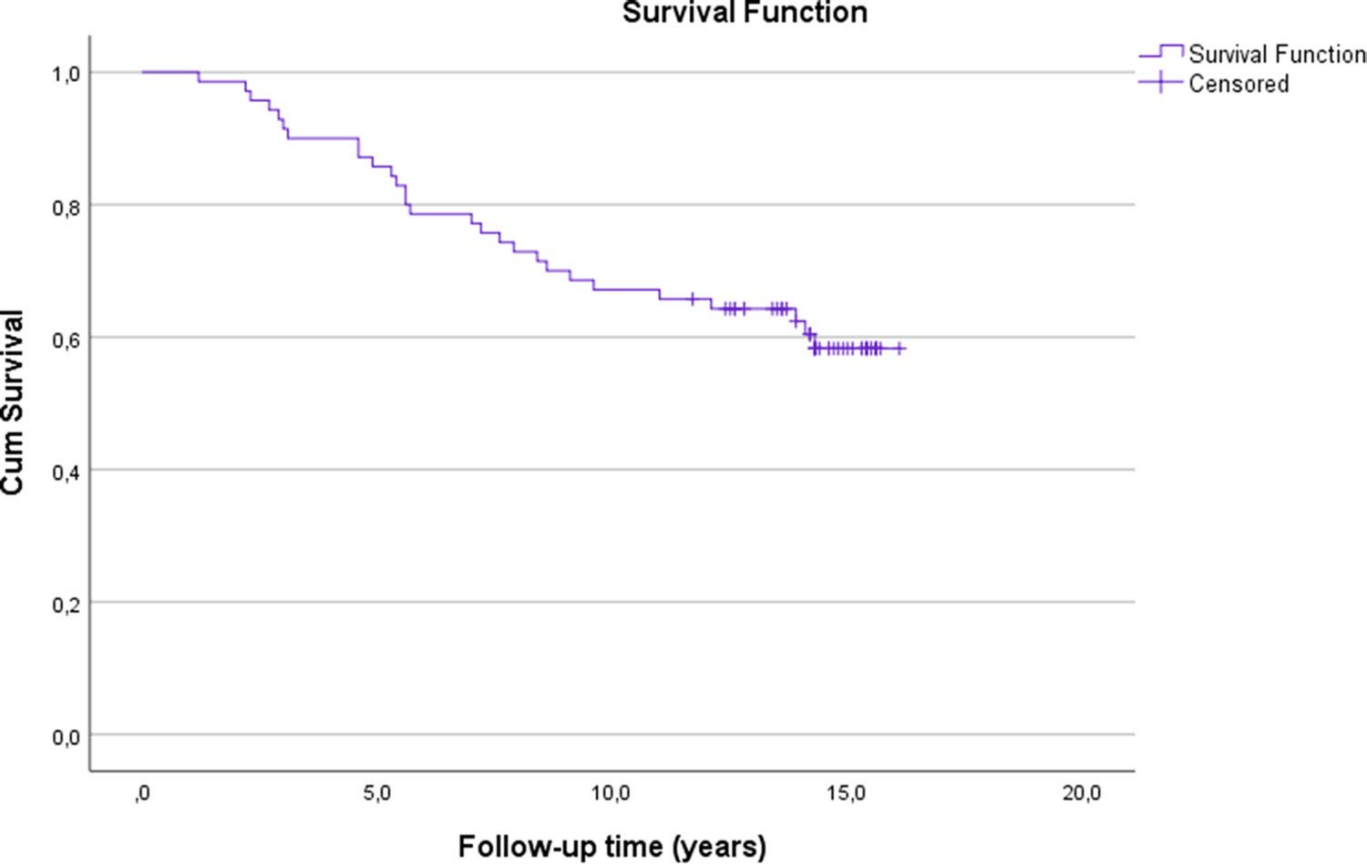
Kaplan–Meier survival analysis of time. The estimates for the cumulative survival with no need for total knee arthroplasty conversion after medial open wedge high tibia osteotomy was 86% at 5-years, 67% at 10-years and 58% at 16.1-years (SE 0.6, CI 95% 11.1–13.5)

**Fig. 4:**
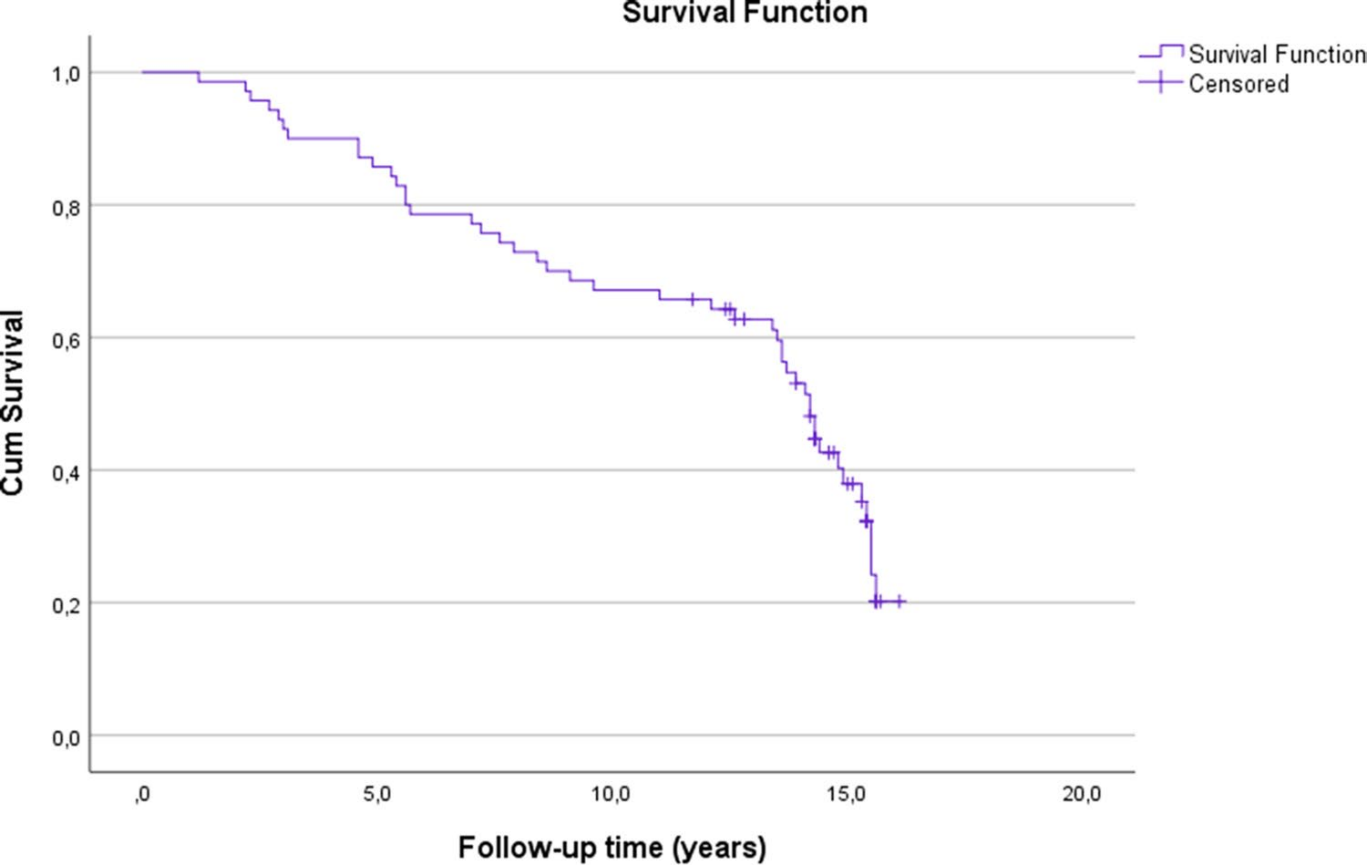
Kaplan–Meier survival analysis of time. The estimates for the cumulative survival with no need for revision due to any reason was 86% at 5-years, 67% at 10-years and 20% at 16.1-years (SE 0.6, CI 95% 10.7–13.0)

**Fig. 5:**
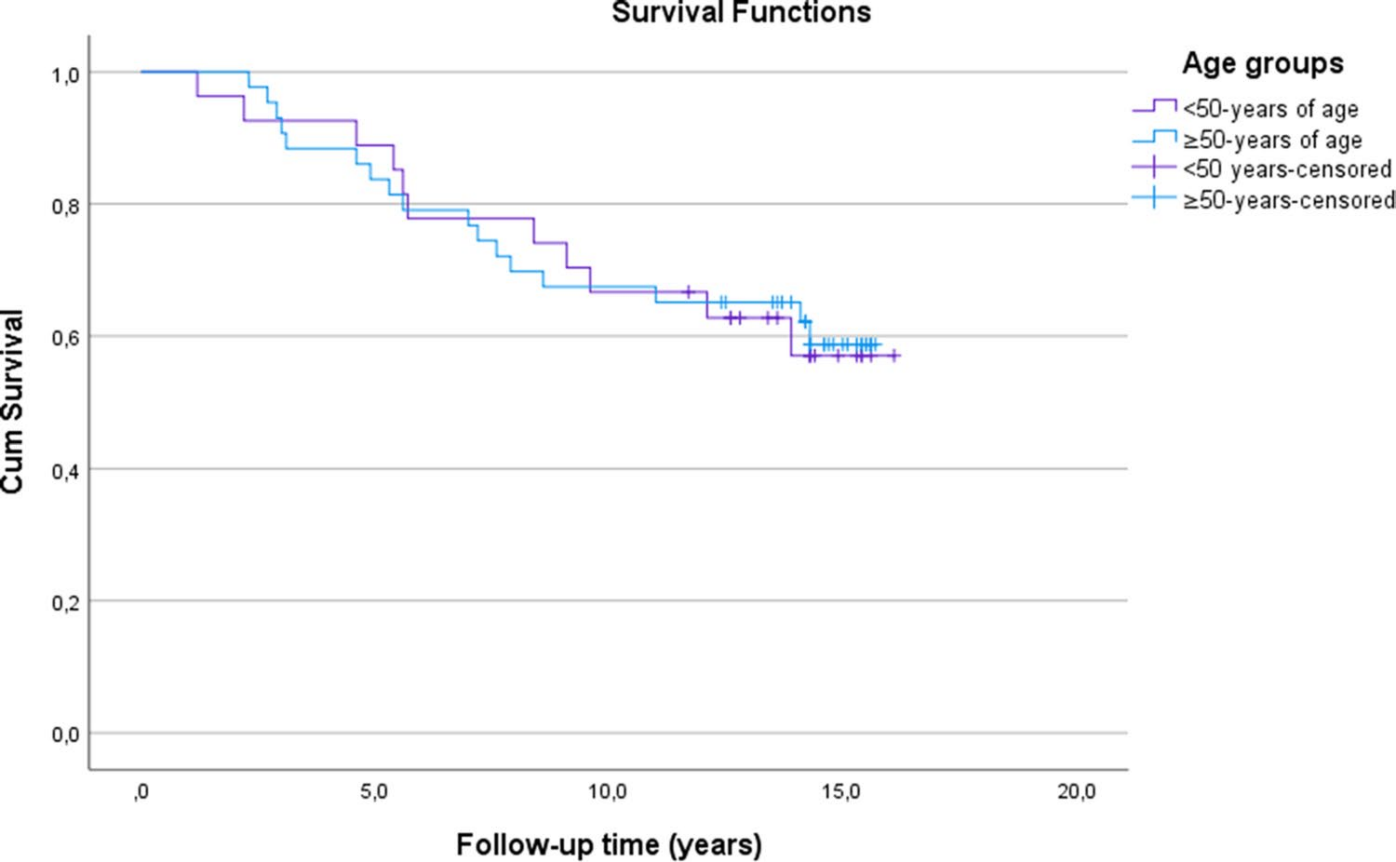
Kaplan–Meier survival analysis of time. The estimates for the cumulative survival with no need for total knee arthroplasty conversion after medial open wedge high tibia osteotomy for patients < 50-years was 89% at 5-years, 67% at 10-years and 57% at 16.1-years (SE 1.0, CI 95% 10.5–14.3) and for patients ≥ 50-years it was 84% at 5-years, 67% at 10-years and 59% at 15.7 years (SE 0.8, CI 95% 10.6–13.6). Log-rank test, *p* = 0.93

**Fig. 6:**
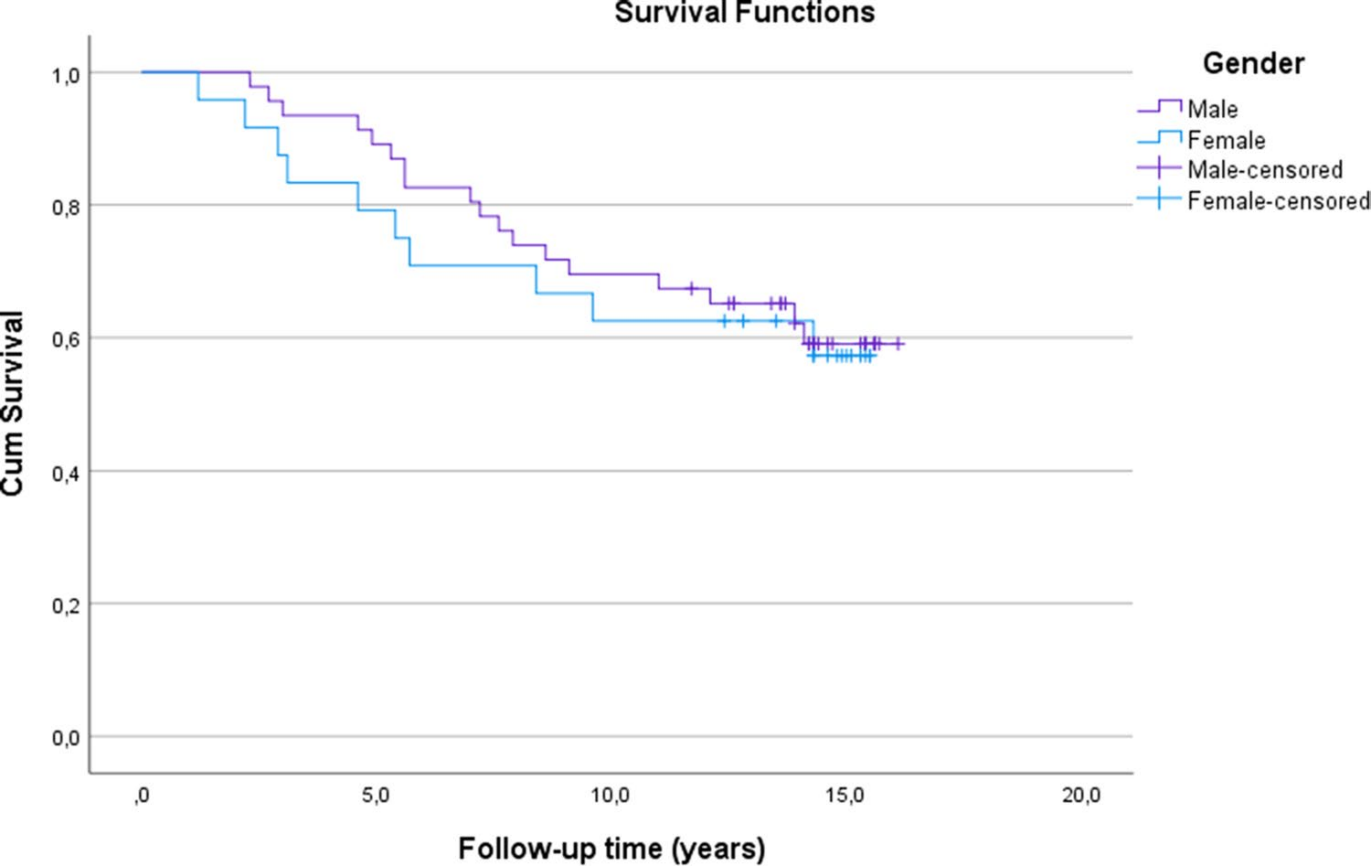
Kaplan–Meier survival analysis of time. The estimates for the cumulative survival with no need for total knee arthroplasty conversion after medial open wedge high tibia osteotomy for females was 79% at 5-years, 63% at 10-years and 57% at 15.5 years (SE 1.1, CI 95% 9.3–13.6) and for males it was 89% at 5-years, 70% at 10-years and 59% at 16.1-years (SE 0.7, CI 95% 11.3–14.0). Log-rank test, *p* = 0.76

The estimates for the cumulative survival with no need for TKA after MOWHTO were 86% at 5 years, 67% at 10 years and 58% at 16.1 years (SE 0.6, CI 95% 11.1–13.5) (Fig. 3). The estimates for the cumulative survival with no need for revision due to any reason were 86% at 5 years, 67% at 10 years and 20% at 16.1 years (SE 0.6, CI 95% 10.7–13.0) (Fig. 4). A subgroup comparison of gender (male:female = 46:24) and age groups < 50 years old (*n* = 27) and ≥ 50 years old (*n* = 43) survival were performed (Figs. 5, 6).

## Discussion

In this study, the cumulative survival of the PP with no need for conversion to TKA after MOWHTO was 86% at 5 years, 67% at 10 years and 58% at 16.1 years. In a similar PP study desing, survival rates of 92–94% at 5 years and 83–94% at 10 years have been reported [6, 15, 21, 22]. Reasons for this study’s poorer survival results might be that patients were older compared to these other similar studies and there were more females in this study [6, 14, 21, 22]. Moreover, comparison of the MOWHTO results is challenging because of the different evaluation systems and techniques used [5, 7, 11–15]. For example, adverse events leading to revision are defined differently, as in some studies, hardware removal due to medial knee pain was not considered as an adverse event at all [5, 7, 13, 15]. In this study, the need for any first-time revision surgery was 38/70 (54%) and revision due to any other reason than conversion to TKA was 14/70 (20%). These results are similar to those shown in previous studies where the complication or major adverse event rate have varied from 37 to 46% [13, 23–26]. The adverse event and intraoperative complication rates in this study were at an acceptable level and were similar to those found in a study by Asik et al. [23]. The need for removal of the PP has been varying from 38 to 55%, which is higher than in this study where this rate was 11/70 (16%) [24, 27]. The definition of the non-union in this study was the absence of radiographic union at 6 months with the persistence of load-dependent pain over the osteotomy. Nonunion leaded to revision in 3% of the cases which is similar to in a larger sample size study where the non-union rate was 3.4% with PP while with more rigid locking plates non-union rates from 0.5 to 3.2% have been reported [28–30].

In previous studies, age > 50 years old and female gender have been associated with a higher risk for revision [14]. In this study, there was not a similar association related to the female gender, but it was found that patients who had a conversion to TKA were statistically significantly older in index MOWHTO surgery compared to those who did not have TKA during follow-up. Previously, it has been found that bone autografts have shown to be superior compared with the use of allografts, and likewise, allografts were superior to bone graft substitutes in a recent systematic review [13]. We did not find statistically significant differences in the use of any osteotomy gap filling in the revision and non-revision group comparison but Cox regression model showed that it was risk factor for revision and conversion to TKA. Previously Slevin et al. [31] suggested that synthetic bone substitutes in MOWHTO could not be recommended. However, due to small sample sizes of subgroups and somewhat unclear indication for use of bone graft or substation, influence of these findings remains unclear in this study.

The BMI was high (mild obesity) in both the revision and non-revision groups, however, the Cox regression model showed that BMI was not a risk factor for revision. Moreover, it has been shown that the influence of BMI in outcomes of HTO is somewhat controversial [14, 25]. It is possible that patients’ high BMI might have affected to the survival of the PP in this study, as plate’s design provides less stable fixation compared to other types of locking plate implants, which might predispose to loss of stable bony fixation and lead to early revision due to loss of correction [13, 24, 32–34] (Fig. 5).

In a recent systematic HTO review, the mean preoperative HKA was 5.7° varus, which is similar to this study (5.9°) [13]. There is a consensus that the mechanical axis in the varus misaligned OA knee should be shifted from the varus into the valgus axis to decrease medial joint space pressure and a 3° valgus HKA should not exceed as an overcorrection may lead to problems [35, 36]. These study results support previous data, as in this study the mechanical axis changed from varus to valgus in all knees with the mean postoperative 1.5° valgus [13, 19]. It has been previously found that there is an indirect association between wedge size and the incidence of a complication [25]. However, in this study this indirect association was not found, as the opening wedge was similar in both the revision and non-revision groups. Loss of angular correction is one of the leading reasons for revision, but this study did not confirm that, as there were no statistically significant differences between pre- and postoperative mechanical axes, MPTA and PPTA changes between the comparisons of the revision and non-revision groups. Furthermore, MPTAs and PPTAs in our study were similar to those found in previous studies, both in the revision and non-revision groups, and there were no statistically significant differences between group comparisons [3, 24–26]. In addition, there were wide ranges of PPTA pre- and postoperatively and due to this, the range of change of PPTA was also wide. One reason for this is that there were few patients with post-trauma arthrosis and some other deformities operated on and them caused this wide range preoperatively and it showed also postoperatively. Moreover, for some of these study patients with severe varus malalignment, a double level osteotomy might have possibly been a better option than just MOWHTO as it normalises the HKA alignment and has shown to lead to good clinical results [37] (Fig. 6).

The strength of this study is its detailed demographic and radiologic analysis of all consecutively operated patients with long-term follow-up. However, this retrospective study design has some inherent limitations, which might have been minimized by a prospective study design. Although all surgeons were experienced, a single surgeon with experience would have been preferable to reduce possible operator-dependent variability [30]. To limit bias, an orthopedic surgeon not involved in patient treatment evaluated the medical records, operative notes, and radiographs. The small sample size of the patients, especially the small female cohort, in the subgroup analysis might be a cofounding factor in the analysis. Furthermore, there were no patient-reported outcome measurements available for this study. Nowadays in general and also in our clinic it is more common technique to use a locking plate concept which provides more rigid fixation and earlier full-weight bearing when compared to the non-locking plates [25, 30, 38–40]. The main reason for this change to use locking plates instead of non-locking plates is their better overall survival [25, 30, 38–40].

## Conclusion

The PP has feasible MOWHTO results with a cumulative survival of 67% at 10 years with no need for conversion to TKA. Many adverse events occurred and revision rate due to any reason was high. Age or gender did not have statistically significant differences in terms of survival.

## Supplementary Information

Below is the link to the electronic supplementary material.

Supplementary file1 (PDF 1225 KB)

## References

[1] Coventry MB (1973). Osteotomy about the knee for degenerative and rheumatoid arthritis: indications, operative techniques and results. J Bone Joint Surg Am.

[2] Insall J, Shoji H, Mayer V (1974). High tibial osteotomy: a 5-year evaluation. J Bone Joint Surg Am.

[3] Paley D, Herzenberg JE, Tetsworth K, McKie J, Bhave A (1994). Deformity planning for frontal and sagittal plane corrective osteotomies. Orthop Clin N Am.

[4] Lobenhoffer P (2014). Importance of osteotomy around to the knee for medial gonarthritis Indications, technique and results. Orthopade.

[5] Amendola A, Bonasia DE (2010). Results of high tibial osteotomy: review of the literature. Int Orthop.

[6] DeMeo PJ, Johnson EM, Chiang PP, Flamm AM, Miller MC (2010). Midterm follow-up of opening-wedge high tibial osteotomy. Am J Sports Med.

[7] W-Dahl A, Robertsson O, Lohmander LS (2012). High tibial osteotomy in Sweden, 1998–2007: a population-based study of the use and rate of revision to knee arthroplasty. Acta Orthop.

[8] Puddu G, Cipolla M, Cerullo G, Franco V, Giannì E (2007). Osteotomies: the surgical treatment of the valgus knee. Sports Med Arthrosc.

[9] Amendola A, Panarella L (2005). High tibial osteotomy for the treatment of unicompartmental arthritis of the knee. Orthop Clin North Am.

[10] Sun X, Wang J, Su Z (2020). A meta-analysis of total knee arthroplasty following high tibial osteotomy versus primary total knee arthroplasty. Arch Orthop Trauma Surg.

[11] Brouwer RW, Huizinga MR, Duivenvoorden T, van Raaij TM, Verhagen AP, Bierma-Zeinstra SMA (2014). Osteotomy for treating knee osteoarthritis. Cochrane Database Syst Rev.

[12] Lobenhoffer P, van Heerwaarden R, Staubli A, Jakob R (2008). Osteotomies around the knee.

[13] Lash NJ, Feller JA, Batty LM, Wasiak J, Richmond AK (2015). Bone grafts and bone substitutes for opening-wedge osteotomies of the knee: a systematic review. Arthroscopy.

[14] van Houten AH, Heesterbeek PJ, van Heerwaarden RJ, van Tienen TG, Wymenga AB (2014). Medial open wedge high tibial osteotomy: can delayed or nonunion be predicted?. Clin Orthop Relat Res.

[15] Polat G, Balcı Hİ, Çakmak MF, Demirel M, Şen C, Aşık M (2017). Long-term results and comparison of the three different high tibial osteotomy and fixation techniques in medial compartment arthrosis. J Orthop Surg Res.

[16] Kellgren JH, Lawrence JS (1957). Radiological assessment of osteoarthrosis. Ann Rheum Dis.

[17] International Cartilage Repair Society. Hyaline cartilage lesion classification system (1998). https://cartilage.org/. Accessed 20 June 2020.

[18] Moreland JR, Bassett LW, Hanker GJ (1987). Radiographic analysis of the axial alignment of the lower extremity. J Bone Joint Surg Am.

[19] Fujisawa Y, Masuhara K, Shiomi S (1979). The effect of high tibial osteotomy on osteoarthritis of the knee. An arthroscopic study of 54 knee joints. Orthop Clin N Am.

[20] Lobenhoffer P, Agneskirchner JD (2003). Improvements in surgical technique of valgus high tibial osteotomy. Knee Surg Sports Traumatol Arthrosc.

[21] Ekeland A, Nerhus TK, Dimmen S, Thornes E, Heir S (2017). Good functional results following high tibial opening-wedge osteotomy of knees with medial osteoarthritis: a prospective study with a mean of 8.3 years of follow-up. Knee.

[22] Orrego M, Besa P, Orrego F, Amenabar D, Vega R, Irribarra L, Espinosa J, Vial R, Phillips V, Irarrázaval S (2020). Medial opening wedge high tibial osteotomy: more than 10 years of experience with Puddu plate technique supports its indication. Int Orthop.

[23] Asik M, Sen C, Kilic B, Goksan SB, Ciftci F, Taser OF (2006). High tibial osteotomy with Puddu plate for the treatment of varus gonarthrosis. Knee Surg Sports Traumatol Arthrosc.

[24] Osti M, Gohm A, Schlick B, Benedetto KP (2015). Complication rate following high tibial open-wedge osteotomy with spacer plates for incipient osteoarthritis of the knee with varus malalignment. Knee Surg Sports Traumatol Arthrosc.

[25] Miller BS, Downie B, McDonough EB, Wojtys EM (2009). Complications after medial opening wedge high tibial osteotomy. Arthroscopy.

[26] Spahn G (2004). Complications in high tibial (medial opening wedge) osteotomy. Arch Orthop Trauma Surg.

[27] Duivenvoorden T, Brouwer RW, Baan A, Bos PK, Reijman M, Bierma-Zeinstra SMA (2016). Comparison of closing-wedge and opening-wedge high tibial osteotomy for medial compartment osteoarthritis of the knee: a randomized controlled trial with a 6-year follow-up. J Bone Joint Surg Am.

[28] Bode G, von Heyden J, Pestka J, Schmal H, Salzmann G, Südkamp N (2015). Prospective 5-year survival rate data following open-wedge valgus high tibial osteotomy. Knee Surg Sports Traumatol Arthrosc.

[29] Han SB, In Y, Oh KJ, Song KY, Yun ST, Jang KM (2019). Complications associated with medial opening-wedge high tibial osteotomy using a locking plate: a multicenter study. J Arthroplasty.

[30] Martin R, Birmingham TB, Willits K, Litchfield R, Lebel ME, Giffin JR (2014). Adverse event rates and classifications in medial opening wedge high tibial osteotomy. Am J Sports Med.

[31] Slevin O, Ayeni OR, Hinterwimmer S, Tischer T, Feucht MJ, Hirschmann MT (2016). The role of bone void fillers in medial opening wedge high tibial osteotomy: a systematic review. Knee Surg Sports Traumatol Arthrosc.

[32] Polyzois D, Stavlas P, Polyzois V, Zacharakis N (2006). The oblique high tibial osteotomy technique without bone removal and with rigid blade plate fixation for the treatment of medial osteoarthritis of the varus knee: medium and long-term results. Knee Surg Sports Traumatol Arthrosc.

[33] Raja Izaham RM, Abdul Kadir MR, Abdul Rashid AH, Hossain MG, Kamarul T (2012). Finite element analysis of Puddu and Tomofix plate fixation for open wedge high tibial osteotomy. Injury.

[34] Stoffel K, Stachowiak G, Kuster M (2004). Open wedge high tibial osteotomy: biomechanical investigation of the modified Arthrex osteotomy plate (Puddu Plate) and the TomoFix Plate. Clin Biomech.

[35] Hernigou P, Medevielle D, Debeyre J, Goutallier D (1987). Proximal tibial osteotomy for osteoarthritis with varus deformity. A 10–13-year follow-up study. J Bone Joint Surg Am.

[36] Lee SJ, Kim JH, Choi W (2021). Factors related to the early outcome of medial open wedge high tibial osteotomy: coronal limb alignment affects more than cartilage degeneration state. Arch Orthop Trauma Surg.

[37] Schröter S, Nakayama H, Yoshiya S, Stöckle U, Ateschrang A, Gruhn J (2019). Development of the double level osteotomy in severe varus osteoarthritis showed good outcome by preventing oblique joint line. Arch Orthop Trauma Surg.

[38] van Wulfften Palthe AFY, Clement ND, Temmerman OPP, Burger BJ (2018). Survival and functional outcome of high tibial osteotomy for medial knee osteoarthritis: a 10–20-year cohort study. Eur J Orthop Surg Traumatol.

[39] Pape D, Kohn D, van Giffen N, Hoffmann A, Seil R, Lorbach O (2013). Differences in fixation stability between spacer plate and plate fixator following high tibial osteotomy. Knee Surg Sports Traumatol Arthrosc.

[40] Agneskirchner JD, Freiling D, Hurschler C, Lobenhoffer P (2006). Primary stability of four different implants for opening wedge high tibial osteotomy. Knee Surg Sports Traumatol Arthrosc.

