# Correction to: Long-term follow-up results of medial opening wedge high tibia osteotomy with a pre-countered non-locking steel plate

**DOI:** 10.1007/s00402-021-03996-9

**Published:** 2021-06-28

**Authors:** Simo S. A. Miettinen, Hannu J. A. Miettinen, Jussi Jalkanen, Antti Joukainen, Heikki Kröger

**Affiliations:** 1 Department of Orthopaedics, Traumatology, and Hand Surgery, Kuopio University Hospital, P.O. Box 1777, 70211 Kuopio, Finland; 2 Faculty of Health Sciences, University of Eastern Finland, Yliopistonranta 1, 70210 Kuopio, Finland

## Correction to: Archives of Orthopaedic and Trauma Surgery 10.1007/s00402-021-03927-8

The original version of this article unfortunately contained a mistake. Figures 5 and 6 captions were interchanged.

**Fig. 5:**
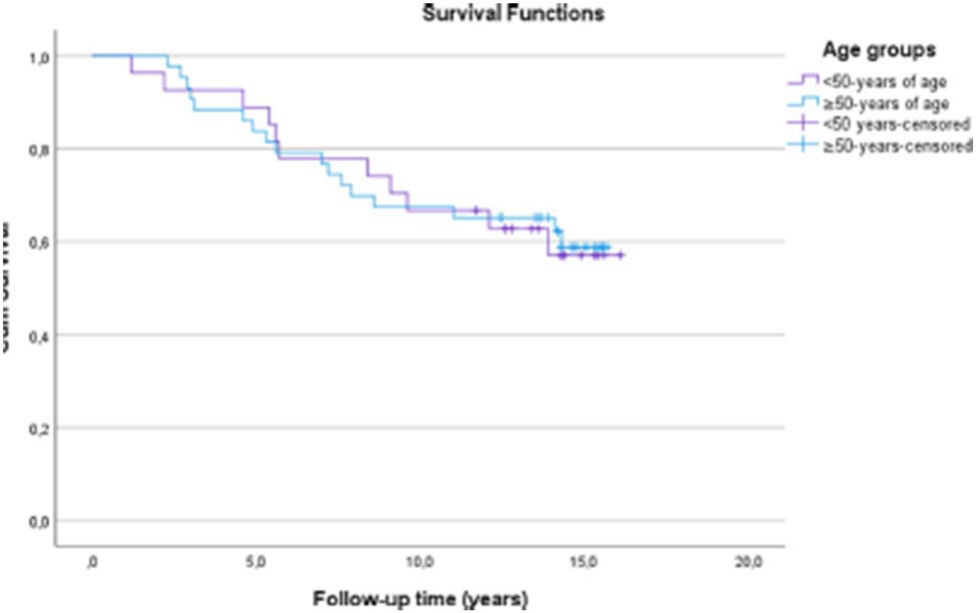
Kaplan–Meier survival analysis of time. The estimates for the cumulative survival with no need for total knee arthroplasty conversion after medial open wedge high tibia osteotomy for patients < 50-years was 89% at 5-years, 67% at 10-years and 57% at 16.1-years (SE 1.0, CI 95% 10.5–14.3) and for patients ≥ 50-years it was 84% at 5-years, 67% at 10-years and 59% at 15.7 years (SE 0.8, CI 95% 10.6–13.6). Log-rank test, *p* = 0.93

**Fig. 6:**
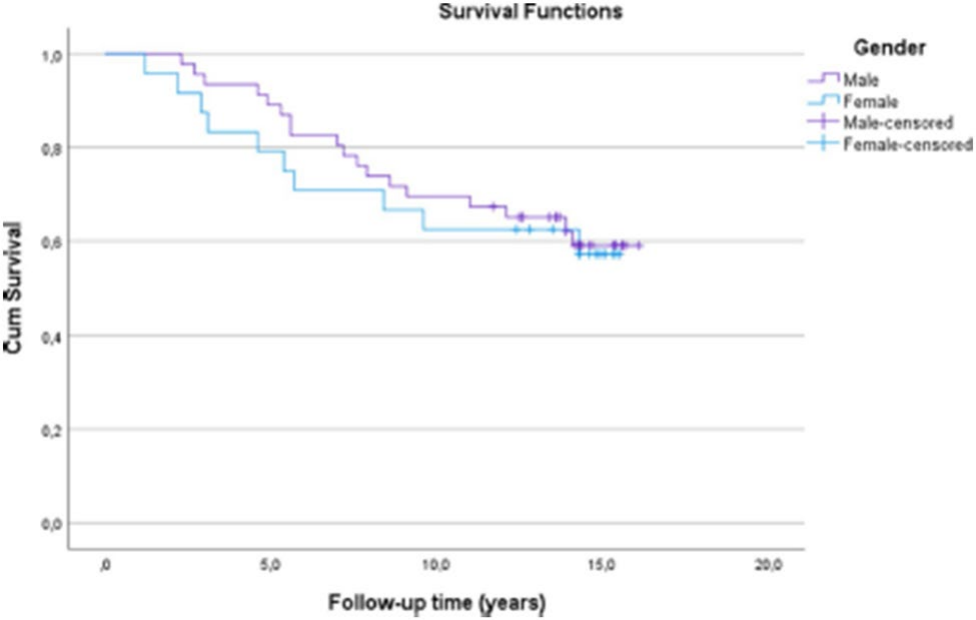
Kaplan–Meier survival analysis of time. The estimates for the cumulative survival with no need for total knee arthroplasty conversion after medial open wedge high tibia osteotomy for females was 79% at 5-years, 63% at 10-years and 57% at 15.5 years (SE 1.1, CI 95% 9.3–13.6) and for males it was 89% at 5-years, 70% at 10-years and 59% at 16.1-years (SE 0.7, CI 95% 11.3–14.0). Log-rank test, *p* = 0.76

The corrected captions with figures are given in the following page.

The original article has been corrected.

